# Surgical treatment of severe epistaxis: an eleven-year experience

**DOI:** 10.5935/1808-8694.20130011

**Published:** 2015-10-14

**Authors:** Paulo Saraceni Neto, Leonardo Mendes Acatauassu Nunes, Luis Carlos Gregório, Rodrigo de Paula Santos, Eduardo Macoto Kosugi

**Affiliations:** ENT at UNIFESP-EPM (Fellow in Rhinology at UNIFESP-EPM); ENT at UNIFESP-EPM (Assistant Physician in the Rhinology Sector at UNIFESP-EPM); PhD in Medicine from UNIFESP-EPM (Head of the Department of ENT & HNS at UNIFESP-EPM); PhD in Sciences from UNIFESP-EPM (Head of the Rhinology Sector at UNIFESP-EPM); PhD in Sciences from UNIFESP-EPM (Coordinator of the Rhinology Fellowship and Lead Preceptor in the ENT Medical Residency at UNIFESP-EPM). Division of Rhinology, Department of Otorhinolaryngology and Head and Neck Surgery UNIFESP

**Keywords:** epistaxis, natural orifice endoscopic surgery, otorhinolaryngologic surgical procedures

## Abstract

Epistaxis is one of the most prevalent emergencies in ENT practice, and its surgical treatment is part of the routine at services for emergency care, especially in cases refractory to clinical procedures.

**Objective:**

To analyze the profile of patients and the results this service has had in the surgical treatment of epistaxis for the last 11 years.

**Method:**

Data from 98 patients submitted to surgery for epistaxis between 2000 and 2011 were analyzed retrospectively.

**Results:**

Most in the sample were males, and mean age was around 46 years. Hypertension was identified in 58% of patients, and most events occurred during fall and winter. The re-bleeding rate was 13.27%.

**Conclusion:**

This study concluded that the surgical treatment for epistaxis, when indicated, had good success rates and low incidence of complications. In our service, it remains as the gold-standard procedure for nosebleeds refractory to initial management measures.

## INTRODUCTION

Epistaxis is one of the most frequent emergencies in ENT care. The prevalence rate of this potentially risky occurrence in the general population is of approximately 12%^1^. However, only 10% of the patients with epistaxis seek medical care, and the initial measures taken to treat these subjects usually suffice in handling nosebleeds. Among such measures are: keeping the airways patent, attaining hemodynamic stability, and packing the nose - an effective procedure in most cases, but unlike many think, a method that introduces considerable risk of morbidity^2^. Yet, approximately 1% of the patients require surgical intervention to manage epistaxis^3^^,^^4^.

Several procedures to treat epistaxis refractory to clinical management have been described in the literature, such as irrigation with warm water, chemical and electrical cauterization of the nasal mucosa, arterial embolization, open and endoscopic artery ligation surgery^5^, ^6^, ^7^. The ligation of the distal arterial branches has been performed more recently to prevent direct and antegrade circulation of the carotid system that irrigates the nasal cavity and known to have plenty anastomoses. Consequence, the ligation of the maxillary or external carotid artery have been less used due to reduced efficacy and increased morbidity^6^^,^^8^. The vessels most frequently ligated to that end are the sphenopalatine and anterior ethmoidal arteries.

The sphenopalatine artery ligation procedure was first described by Prades in 1970 through microsurgery. Only in 1992, with the development and refinement of nasal endoscopic surgery, did Budrovich & Saetti report the use of an endoscope to perform the procedure^8^. The anterior ethmoidal artery ligation technique is older. It was described in 1946 by Weddell, and was performed through external access via Lynch incision^3^. Potential consequences of the external access approach such as scars, edema, and facial ecchymosis led to the study and development of endoscopic techniques to approach this artery^4^.

This study aimed to present data from the ENT Residency Program regarding the surgical management of severe epistaxis performed between June 2000 and June 2011.

## METHOD

This retrospective longitudinal study included the review of the medical charts of patients seen at the ENT emergency care unit for severe epistaxis and treated surgically between June 2000 and June 2011. Severe epistaxis was defined as a life-threatening episode of nasal bleeding, usually of posterior origin, requiring immediate anteroposterior nose packing to attain hemodynamic stability^9^^,^^10^. All patients treated endoscopically for severe epistaxis through sphenopalatine and/or anterior ethmoidal artery ligation were included. There were no exclusion criteria. This study was approved by the institution's Research Ethics Committee and given permit 87081.

The patients seen for epistaxis at our service were submitted to the initial emergency protocol, which included airway and respiratory care, along with hemodynamic stabilization procedures. Then, they were offered specific care for epistaxis such as suction of clots and administration of topical vasoconstrictors to identify the sites of bleeding and elect the best course of therapy among the options of chemical cauterization with 70% trichloroacetic acid and anterior or anteroposterior nose packing. The patients submitted to surgery were the ones who still bled in spite of the nose packing, who bled soon after nose packing removal, and those submitted to anteroposterior nose packing without clinical contraindication for surgery.

Surgery was carried out with the patients under general anesthesia by third-year medical residents supervised by medical doctors from the Rhinology Sector or attending physicians from the ENT emergency care unit. Ligation and/or cauterization of the sphenopalatine artery branches was carried out by transnasal endoscopy. Cauterization of the anterior ethmoidal artery was preferentially performed through the external approach (Lynch incision) with the aid of an endoscope, and occasionally by transnasal endoscopy. In our service we approach the sphenopalatine artery or the sphenopalatine and anterior ethmoidal arteries, depending on the site of bleeding.

The following data were considered in the definition of the profile of our patients: age, gender, and present comorbidities. Bleeding-related characteristics were also considered, such as side, possible etiology, admission blood tests (hemoglobin, hematocrit, and platelet count), and need for blood transfusion. The following were considered in regards to surgery: date of the procedure, approached artery, number of approached branches of the sphenopalatine artery, time of surgery, complications, surgery effectiveness, and failure. The seasonality of cases of severe epistaxis was also analyzed.

## RESULTS

The charts of 109 patients submitted to surgery for severe epistaxis between June 2000 and June 2011 were included. Seven charts were excluded for lack of information and another four subjects submitted to nose packing under general anesthesia were not included. The characteristics of the patients enrolled in the study are shown on Table 1.

**Table 1:** Characteristics of enrolled patients.

Data	Unit	Value	
Age	Years	Mean	46.68
		SD	15.28
		Minimum	13
		Median	48
		Maximum	81
Gender	N (%)	Male	70 (71.43)
		Female	28 (28.57)
Comorbidities	N (%)	SHT	57 (58.16)
		Chronic alcohol abuse	10 (10.2)
		DM	7 (7.14)
		Chronic use of ASA	5 (5.1)
		Liver disease	4 (4.08)
		CKD	3 (3.06)
		NHL	2 (2.04)
		Arrhythmia	2 (2.04)
		Hypothyroidism	2 (2.04)
		Heart disease	1 (1.02)
		Thrombocytosis	1 (1.02)

N: number; %: percentage; SD: standard deviation; SHT: systemic hypertension; DM: diabetes mellitus; ASA: acetylsalicylic acid; CKD: chronic kidney disease; NHL: non-Hodgkin's lymphoma.

The characteristics related to bleeding can be seen on Table 2.

**Table 2:** Features of epistaxis.

Data	Unit	Value	
Side	N (%)	Right	48 (48.98)
		Left	35 (35.71)
		Bilateral	15 (15.31)
Location	N (%)	Anterior	14 (14.29)
		Posterior	35 (35.71)
		Not identified	49 (50)
Etiology	N (%)	Undefined	64 (65.31)
		Hypertensive peak	19 (19.39)
		Nose surgery FU	5 (5.1)
		Trauma	5 (5.1)
		Coagulopathy	4 (4.08)
		Nasal tumor	2 (2.04)
		Rendu-Osler-Weber	1 (1.02)

N: number; %: percentage; FU: follow-up.

Blood test and transfusion data can be verified on Table 3.

**Table 3:** Blood transfusion characteristics.

Data	Unit	Value	
Hemoglobin	g/dL	Mean	11.42
		SD	2.75
		Minimum	5.6
		Median	11.1
	%	Maximum Mean	16.3
Hematocrit	%	Mean	33.82
		SD	8.2
		Minimum	12.2
		Median	33.05
	N/mL	Maximum Mean	49.5
Platelet count	N/mL	Mean	241.779,41
		SD	120.787,32
		Minimum	42.000
		Median	228.500
		Maximum	972.000
Blood transfusion	N (%)	Yes	21 (21.43)
		No	77 (78.57)
Transfusion patients	PRBC units	Mean	2.81
		SD	1.12
		Minimum	2
		Median	2
		Maximum	6

N: number; %: percentage; SD: standard deviation; PRBC: packed red blood cells.

The 98 patients enrolled in the study underwent surgery under general anesthesia, and had solely their sphenopalatine arteries or their sphenopalatine and anterior ethmoidal arteries approached, on one or both sides. Some patients had more than one artery approached. The data on the first procedure the patients underwent can be seen on Table 4.

**Table 4:** Characteristics of initial surgical approach.

Data	Values N (%)
Approached arteries	
Sphenopalatine 1 branch	93 (86.11)
Sphenopalatine 2 branches	7 (6.47)
Sphenopalatine 3 branches	1 (0.95)
Anterior Ethmoidal	7 (6.47)
Total	108 (100)
Operated patients	
Success	85 (86.73)
Failure	13 (13.27)
Total	98 (100)

N: number; %: percentage.

Epistaxis was successfully managed in 85 cases (86.73%). Six (46.15%) of the 13 patients with failed surgery bled within 24 hours of surgery. Table 5 shows what was done for the 13 patients whose first procedures failed.

**Table 5:** Conduct in failed surgery.

Conduct	N (%)
Nose packing	4 (30.77)
Sphenopalatine Revision	1 (7.69)
Anterior ethmoidal + Sphenopalatine Revision	8 (61.54)
Total	13 (100)

N: number; %: percentage.

In the 13 patients with failed first surgery, only one branch of the sphenopalatine artery was found. More anterior ethmoidal artery ligations were indicated after the failure of the first procedure (eight of the nine re-operated patients) than as a first procedure (seven of 98 operated patients). Indication of anterior ethmoidal artery surgery was significantly higher in reoperations, as seen on Table 6.

**Table 6:** Anterior ethmoidal artery.

Time of surgery	N (%)
Initial approach	7 (46.67)
Approach after failure	8 (53.33)
Total	15 (100)

N: number; %: percentage.

Hospitalization and surgery times are shown on Table 7.

**Table 7:** Length of hospitalization and surgery.

Duration	Value Mean	
Hospitalization (days)	Mean	3.38
	SD	4.53
	Minimum	1
	Median	2
	Maximum	41
	Mode	2
Surgery (minutes)	Mean	113.48
	SD	44.31
	Minimum	20
	Median	110
	Maximum	250

SD: standard deviation.

**Graph 1:**
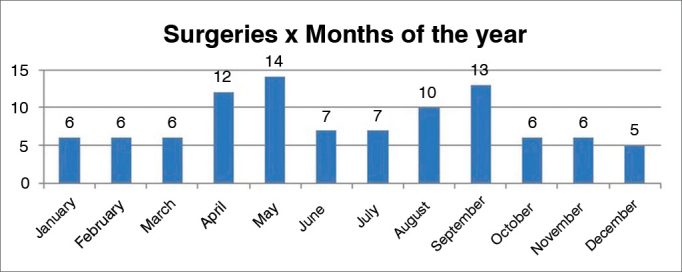
Distribution of surgical procedures per month.

**Graph 2:**
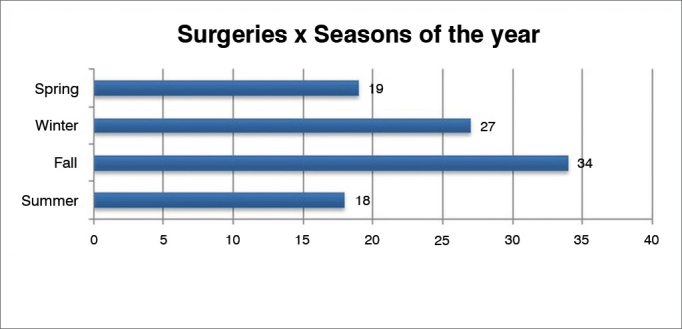
Distribution of surgical procedures per season.

The distribution of procedures and months in which they were performed can be seen on Graph 1, Graph 2.

Four complications (4.08%) were recorded. Two (2.04%) related to surgery (septum perforation and amaurosis), one (1.02%) to anesthesia (death by acute myocardial infarction during anesthesia recovery), and one (1.02%) to hospitalization (hospital-acquired pneumonia).

## DISCUSSION

The ninety-eight surgical patients enrolled in this study represent a significant series when compared to other papers in the literature^2^^,^^3^^,^^6^^,^^11^^,^^12^. Patients with severe epistaxis are usually between the third and eighth decade of life, and the most numerous age group comprehends subjects in their seventies and eighties^2^^,^^3^^,^^6^^,^^8^. The mean age of the subjects in our study was lower (46 years), probably a reflection of the fact that endoscopic surgery was offered to all patients with anteroposterior nose packing, and not only to cases in which surgery failed. There is a slight predominance of males in studies on epistaxis^1^, ^2^, ^3^^,^^5^. In our study, males prevailed by a ratio of 2.5:1.

The side of bleeding has been recently discussed, and a predilection for right epistaxis - albeit without statistical significance - was observed in a group of 326 patients^11^. Our study presented similar results, as 48.98% of the patients had right nasal fossa involvement (n = 48), 35.71% had left nosebleeds (n = 35), and 15,.31% had bilateral epistaxis (n = 15). In terms of the site of bleeding - anterior, posterior, or diffuse - the charts revealed that posterior bleeds were more frequent, with 35.71% (n = 35), against 14.29% (n = 14) of anterior nosebleeds. The remaining 50% did not have defined bleed sites or were described as ‘diffuse'. Most nosebleeds are known to occur in anterior sites associated with Kiesselbach's plexus and account for approximately 85% of all cases^1^^,^^11^, but our study indicated a preponderant role of posterior bleeds in severe epistaxis.

The etiology of severe epistaxis cannot be defined for all cases. There is confusion between predisposing and aggravating factors, and they do not necessarily establish a causal link to bleeding, which adds to the difficulty of comparing this study to others^8^^,^^11^. Clear nosebleed etiology is established for only 15% of the subjects, and idiopathic cases are more prevalent^8^. In our study, etiology was defined in 34.69% (n = 34) of the cases of epistaxis. The diagnoses included: hypertensive peaks (19.39%), bleeds following recent nose and paranasal sinus surgery (5.1%), trauma (5.1%), coagulopathy (4.08%), nasal tumors (2.04%), and hereditary hemorrhagic telangiectasia (1.02%). The treatment for severe epistaxis of known etiology must include, whenever possible, solutions for the cause of the problem; for example, the management of hypertensive peaks and reversal of coagulopathy^13^. Coagulopathy has been traditionally seen as a contraindication to surgery, but the ligation or cauterization of the branches of the sphenopalatine artery have provided for satisfactory results in these cases^9^. In epistaxis caused by nasal tumors, the role of arterial ligation alone is more limited. Treatment of such cases usually involves the resection of the tumor that originated the nosebleed, in addition to artery ligation^13^. Artery ligation is not the main option to treat hereditary hemorrhagic telangiectasia, and was used in one of our patients solely because of severe epistaxis, and not for the underlying condition.

The most prevalent comorbidity found in the studied population was systemic hypertension, seen in 58.16% of the patients (n = 57), as similarly reported by other authors^3^^,^^8^. Despite the difficulties establishing a causal correlation between systemic hypertension and epistaxis -and even more so severe epistaxis - it is important to stress that the prevalence rate seen in the studied population was considerably higher than the rates observed in the Brazilian population, which range around an estimated 20%^14^. Other aggravating factors and conditions related to nosebleeds include diabetes mellitus, chronic alcohol abuse accompanied or not by alcoholic liver disease, smoking, heart arrhythmias, tumors, use of antiplatelet drugs, anticoagulants, and illicit drugs such as cocaine. These factors have also been cited by other authors in varying degrees of relevance, although never at the same level of importance as systemic hypertension^1^^,^^2^^,^^11^.

This study also assessed the hemodynamic impact of surgical cases of epistaxis. In some cases, this information was essential for surgical indication. It should be noted that the top platelet count seen in our study due to a case of thrombocytosis without clinical repercussions pulled up the mean value of this variable. The variation of blood test results observed in this study was similar to previous reports in the literature^6^^,^^12^. Low platelet count has been considered an isolated risk factor for early rebleed in patients submitted to epistaxis surgery^1^, but our study failed to reproduce such finding. The literature also indicates that 50% of the patients with epistaxis submitted to surgery require blood transfusion^1^^,^^6^^,^^12^, whereas in our study only 21.4% of the patients (n = 21) needed transfusion. A mean 2.8 units of packed red blood cells were administered to patients requiring transfusion.

The number of arterial branches in the sphenopalatine foramen has also been a topic of controversy. A study done on cadavers submitted to nasal endoscopy showed that 67.21% of the nasal fossae had one arterial trunk leaving the sphenopalatine foramen, 21.31% had two branches, and 11.47% had three^15^. In our study, one branch in the sphenopalatine foramen was seen in 86.11% of the nasal fossae. The difference against the literature may be explained by the greater difficulty dissecting the sphenopalatine foramen during surgery in live patients than in fresh cadavers.

Success rates were quite good, and rebleeds were seen in 13.27% (n = 13) of the patients. Most patients in this situation (n = 8) were reoperated and had the hemostasis of the sphenopalatine artery territory revised and the anterior ethmoidal artery (AEA) ligated. All rebleeds had one branch of the sphenopalatine artery identified. A study by Holzmann showed similar results, as higher rebleed rates were seen in cases in which only the lateral branches of the sphenopalatine artery were occluded^2^. This data supports the need to thoroughly explore the sphenopalatine foramen, in an active search for all branches arising from it. Holzmann also reported increased success rates when dissection and occlusion of the septal branches of this vessel were performed. Rebleeds occurring in those cases can be accounted for by the antegrade flow that occurs mainly in the septal territory, an area with many anastomoses between different arterial systems^2^.

AEA ligation to manage epistaxis is still controversial. Although many authors believe that this vessel should be approached in the first procedure along with the sphenopalatine artery and its branches, most papers show this technique being used to manage rebleed cases^2^^,^^16^. The improvement of the techniques used to endoscopically approach the AEA may facilitate the indication of AEA ligation as the procedure of choice. The approach in use today includes external access using the Lynch incision which, aside from a scar, may leave other complications such as facial edema and epiphora - possibly the reasons why surgeons are no longer opting to indicate this procedure initially^4^.

Time of hospitalization in the revision cases shows that, in addition to being effective, surgery does not lead to prolonged hospital stays. The mean hospital stay length was three days, as also reported in the literature^8^^,^^16^. The mean time of hospitalization was overestimated in our study, as one patient with complications had to stay in for 41 days. The mode, however, was two days of hospitalization. Surgery mean length was one hour and 53 minutes.

The influence of weather conditions in the incidence of epistaxis has been analyzed by a number of authors^17^^,^^18^. However, controversy and contradicting results still abound. In our sample, more cases were seen in the fall and winter months. However, one cannot state that such finding is truly related to the weather. An inventory of meteorological data for each year of the study would have to be analyzed along with the variations in temperature and humidity before statements of this sort could be made. The observed complication rate of 4.08% shows how safe the employed procedures were, mainly if one takes into account that only half of these complications were related to surgery. The main of them, a case of amaurosis, may be explained by the lack of experience of the surgeon while performing electrocoagulation in an area too close to the nerve bundles of the orbit.

## CONCLUSION

This study presented the 11 years of experience of our service in surgically managing severe epistaxis with a success rate of 86.73%. In our series, careful identification of all arterial branches in the sphenopalatine foramen and early anterior ethmoidal artery ligation were identified as contributing factors to higher success rates.
